# Associations of airway tree to lung volume ratio on computed tomography with lung function and symptoms in chronic obstructive pulmonary disease

**DOI:** 10.1186/s12931-019-1047-5

**Published:** 2019-04-18

**Authors:** Naoya Tanabe, Susumu Sato, Tsuyoshi Oguma, Hiroshi Shima, Atsuyasu Sato, Shigeo Muro, Toyohiro Hirai

**Affiliations:** Department of Respiratory Medicine, Graduate School of Medicine, Kyoto University, 54 Kawahara-cho, Shogoin, Sakyo-ku, Kyoto, 606-8507 Japan

**Keywords:** Chronic obstructive pulmonary disease, Computed tomography, Airway, Pulmonary function, Emphysema, Symptom

## Abstract

**Background:**

Decreased airway lumen size and increased lung volume are major structural changes in chronic obstructive pulmonary disease (COPD). However, even though the outer wall of the airways is connected with lung parenchyma and the mechanical properties of the parenchyma affect the behaviour of the airways, little is known about the interactions between airway and lung sizes on lung function and symptoms. The present study examined these effects by establishing a novel computed tomography (CT) index, namely, airway volume percent (AWV%), which was defined as a percentage ratio of the airway tree to lung volume.

**Methods:**

Inspiratory chest CT, pulmonary function, and COPD Assessment Tests (CAT) were analysed in 147 stable males with COPD. The whole airway tree was automatically segmented, and the percentage ratio of the airway tree volume in the right upper and middle-lower lobes to right lung volume was calculated as the AWV% for right lung. Low attenuation volume % (LAV%), total airway count (TAC), luminal area (Ai), and wall area percent (WA%) were also measured.

**Results:**

AWV% decreased as the Global Initiative for Chronic Obstructive Lung Disease (GOLD) spirometric grade increased (*p* < 0.0001). AWV% was lower in symptomatic (CAT score ≥ 10) subjects than in non-symptomatic subjects (*p* = 0.036). AWV% was more closely correlated with forced expiratory volume in 1 s (FEV_1_) and ratio of residual volume to total lung capacity (RV/TLC) than Ai, Ai to lung volume ratio, and volume of either the lung or the airway tree. Multivariate analyses showed that lower AWV% was associated with lower FEV_1_ and higher RV/TLC, independent of LAV%, WA%, and TAC.

**Conclusions:**

A disproportionally small airway tree with a relatively large lung could lead to airflow obstruction and gas trapping in COPD. AWV% is an easily measured CT biomarker that may elucidate the clinical impacts of the airway-lung interaction in COPD.

**Electronic supplementary material:**

The online version of this article (10.1186/s12931-019-1047-5) contains supplementary material, which is available to authorized users.

## Background

Chronic obstructive pulmonary disease (COPD) is characterized by expiratory airflow obstruction that is caused by a combination of emphysematous destruction of the parenchyma and disease of the small airways [1]. Because obtaining pathological samples of lung tissue is too invasive to perform in clinical practice, chest computed tomography (CT) has been used to obtain separate estimations of these two pathological lesions [2]. Emphysematous changes measured by CT is associated with worsening of lung function [3] and symptoms [4], frequent exacerbation [5], and poor prognosis [6]. In addition, loss of elastic recoil due to emphysema causes lung hyper-expansion that can also be quantified as total lung volume in CT [7]. The CT-measured total lung volume has been used for evaluating the effects of pharmacological, bronchoscopic, and surgical lung volume reduction, as well as lung growth [8–11].

Although small airways cannot be visualized by CT due to the resolution limitation [3, 12], measurements of the central airway dimensions are informative because these dimensions reflect the histological features of small airway disease [13]. In particular, a small airway lumen area in COPD is an important CT finding that is closely correlated with airflow obstruction, as assessed by forced expiratory volume in 1 s (FEV_1_) [14, 15] and gas trapping, as assessed by residual volume (RV), or its ratio to total lung capacity (TLC) [RV/TLC] [7]. Moreover, these smaller lumens of the airways reduce the number of airways visible on CT, and this impaired visibility is currently assessed as total airway count (TAC) [16], which has been shown to be associated with FEV_1_, dyspnoea, exercise tolerance and future lung function decline [16, 17].

Since the outer wall of the airways is connected with the lung parenchyma, the mechanical properties of the parenchyma affect the behaviour of the airways [18]. This airway-parenchyma interdependence allows the elastic recoil of the lung to maintain airway calibre, indicating that any disease condition inducing loss of elastic recoil could reduce airway calibre [19]. Indeed, histological and microCT analyses have shown that loss of the alveolar attachments to the outer wall of the small airways is closely correlated with lumen narrowing in emphysema [20–22]. A CT study by Diaz et al. [23] also showed that segmental and subsegmental airways are distended during breathing up to the TLC level in healthy lungs, but this distensibility is diminished in hyper-inflated emphysematous lung and further reduction of the lumen area occurs at full inspiration. Moreover, physiological and CT imaging studies have shown that the association between airway and lung sizes is not constant even in healthy subjects [24, 25], and suggested that dysanaptic lung growth that is characterized by an increase in lung size without change in airway size in early life might impair lung function [9, 25], supporting a recent notion that abnormal lung growth potentially affects COPD development [26]. However, little is still known about the interaction between airway and lung sizes on lung function and symptoms in COPD.

It was hypothesized that a reduction in the ratio of airway tree volume to lung volume could be a major determinant of impaired pulmonary function and clinical symptoms in COPD. To test this hypothesis, the present study aimed to establish a novel CT marker, namely, airway volume percent (AWV%), that was defined as a percentage ratio of the airway tree volume to the entire lung volume and to explore the associations of the AWV% with lung function and symptoms assessed by the COPD Assessment Test (CAT).

## Methods

### Ethics

This study was performed in accordance with the Declaration of Helsinki. This human study was approved by the Ethics Committee of Kyoto University (approval No. E182). Written informed consent was obtained from all participants.

### Study subjects

The data used in the present analysis were based on those from a prospective observational study performed at Kyoto University [27, 28]. The inclusion criteria were (1) a smoking history of at least 20 pack-years, (2) COPD diagnosis, and (3) no history of lung resection surgery or other lung diseases, such as bronchial asthma or interstitial lung disease. All male patients who underwent chest inspiratory CT scans and completed lung function testing including spirometry, lung volumes and diffusion capacity measurements, and a CAT questionnaire during an exacerbation-free period were enrolled at Kyoto University Hospital from April 2011 to April 2014. Lung function was measured with a Chestac-65 V (Chest MI Corp., Tokyo, Japan), and chest CT scans were performed with an Aquilion 64 scanner (Toshiba; Tokyo, Japan). Calibration of the CT scanner was routinely performed with air and water phantoms, and the scanning conditions were as follows: 0.5-mm collimation, 500-millisecond scan time, 120 peak kilovoltage, and auto-exposure control. Reconstruction was performed with a high spatial frequency algorithm (FC56) as previously reported [27].

### CT analysis

#### Airway volume percent (AWV%)

To evaluate the volume of the airways visible on CT images (Additional file 1: Figure S1A), the entire airway tree was automatically segmented without any manual editing using a SYNAPSE VINCENT volume analyser Ver5.3 (FUJIFILM Medical, Tokyo, Japan). The segmented airway trees were exported as DICOM files and further segmented into portions of the right upper lobe (RUL), right middle-lower lobe (RMLL), and the remaining lobes with ITK-SNAP software (Additional file 1: Figure S1B) [29] . The airway tree in the left lung was not used because segmentation of this area could be affected by cardiac motion artefacts. The volumes of the RUL and RMLL were added to obtain the total airway volume in the right lung (AWV). Right lung volume (rLV) was also measured, and the airway volume percent (AWV%) was calculated with the following formula: AWV% = 100 * AWV / rLV using custom software. Because the luminal size of the central airways was affected by the natural size of the lungs, the predicted total lung capacity (pTLC) was calculated [30] for adjustment of the AWV and right lung volume.

#### Total airway count (TAC)

Following the segmentation of the airway trees in the RUL and RMLL, these segmented trees were skeletonized to obtain their centre lines using custom software. Each branch of the centre line between branching points was labelled. Based on this labelling, the airway trees in the RUL and RMLL were separated into branches, and all the branched trees were labelled (Additional file 1: Figure S1C). Corrections of labels were performed manually when necessary.

#### Luminal area (Ai) and wall area percent (WA%)

Using the custom software, the lumen and wall of the right apical and lower posterior segmental and sub-segmental airways (RB1 and RB10 paths) were segmented with the full-width half-maximum principle as reported previously [3, 31]. The lumen area (Ai) and wall area (WA) were measured, and then, the wall area percent (WA%) was calculated using the following formula: 100*WA / (sum of Ai and WA). The average Ai and WA% from RB1 and RB10 were used for the present analyses.

#### Low attenuation volume percent (LAV%)

Using the SYNAPSE VINCENT volume analyser, lung volume (CT-TLV) and the percentage ratio of voxels < − 950 HU to the entire lung voxels, namely, the low attenuation volume percent (LAV%), for both lungs and the right lung alone were measured [8, 16, 27, 32–34].

### Statistics

The data are expressed as the mean ± standard deviation (SD). Pearson correlation analysis was used to evaluate associations among CT measures and pulmonary function. Multivariate linear regression analysis including CT indices, age, body mass index (BMI), and smoking pack-years as independent variables and each pulmonary function value as dependent variables was performed. Statistical analysis was performed with R [35]. A *p*-value less than 0.05 was considered statistically significant.

## Results

**Fig. 1:**
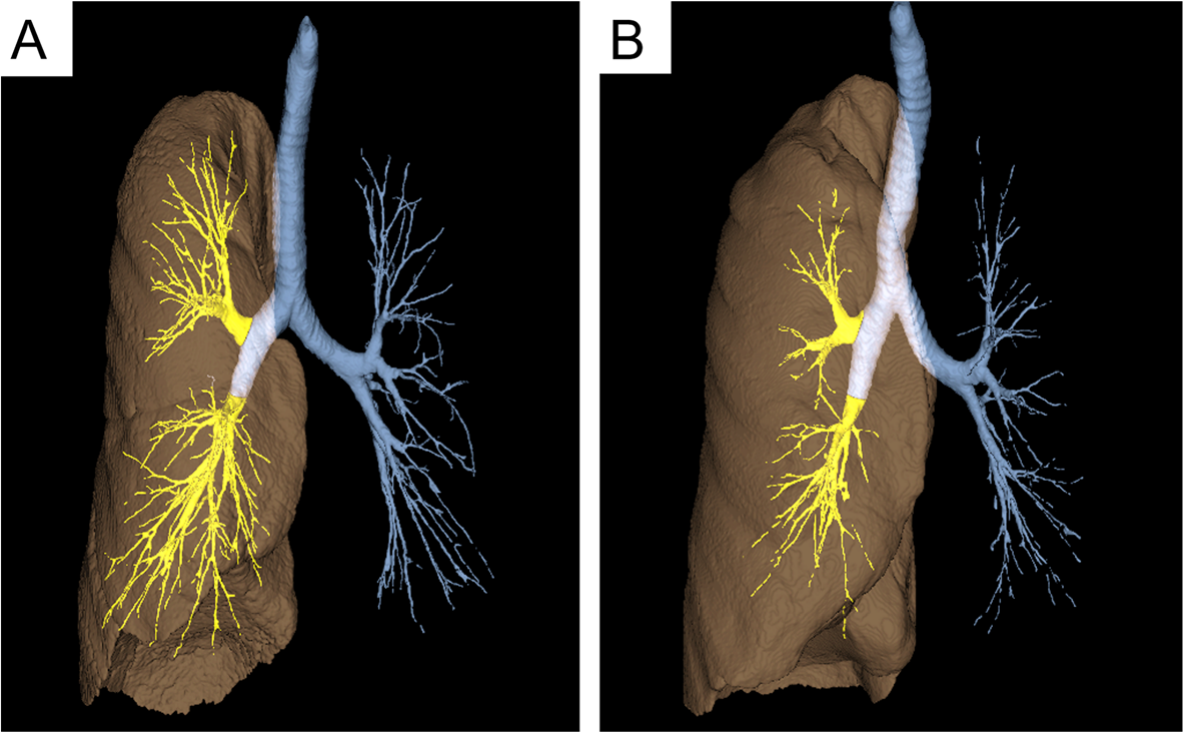
Representative 3D renderings of airway tree and right lung in 2 COPD cases. A percentage ratio of airway tree volume in the right upper and middle-lower lobes (AWV, highlighted by yellow colour) to the right lung volume (rLV, brown colour), namely, the airway volume percent (AWV%), was compared between COPD subjects with mild airflow obstruction (Case A, %FEV_1_ = 81%, height = 167 cm) and with severe airflow obstruction (Case B, %FEV_1_ = 32%, height = 167 cm). Compared to Case A (AWV = 18 ml, rLV = 2519 ml), Case B showed smaller airway tree volume and larger lung volume (AWV = 9 ml, rLV = 2998 ml), which resulted in substantially lower AWV%

The basic data of study subjects are summarized in Table 1. Figure 1 shows representative 3D renderings of the airway tree and the right lung in COPD subjects with mild and severe airflow obstruction (cases A and B, %FEV1 = 81 and 31%, respectively). Although there was no difference in height (both 167 cm), the AWV was smaller (9 vs 18 ml) and right lung volume was larger (2998 vs 2519 ml) in case B than Case A, which made a substantial difference in the AWV% between the two cases.

**Table 1:** Patient demographics (*n* = 147)

Age	71 ± 9
Sex	All male
BMI	22 ± 3
Smoking pack-years	63 ± 35
FEV_1_ (% predicted)	61 ± 20
FEV_1_/FVC (%)	51 ± 13
RV/TLC (%)	42 ± 7
TLC (% predicted)	96 ± 13
D_LCO_ (% predicted)	53 ± 14
CAT score ≧10 [n (%)]	81 (55%)
LAV%	29 ± 9
WA% (segmental airways)	58 ± 5
WA% (sub-segmental airways)	64 ± 4
TAC (right lung)	212 ± 51

Data are expressed as the mean ± SD. *BMI* body mass index, *FEV*_*1*_ forced expiratory volume in 1 s, *FVC* forced vital capacity, *RV/TLC* residual volume / total lung capacity, *D*_*LCO*_ diffusion capacity, *CAT* COPD Assessment Test, *LAV%* low attenuation volume percent, *WA%* wall area percent, *TAC* total airway count.

**Fig. 2:**
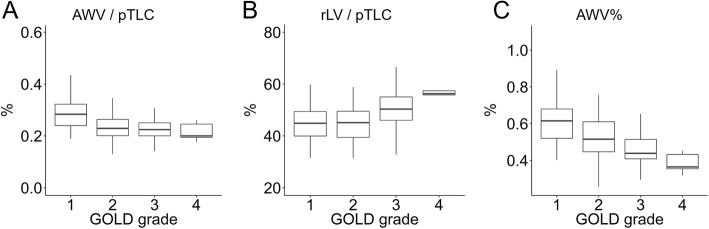
Airway and lung measures and severity of COPD (A and B) The airway wall volume (AWV) and right lung volume (rLV) are adjusted by the predicted total lung capacity (pTLC). (C) The airway wall volume percent (AWV%) was the percentage ratio of the AWV to right lung volume. Statistical analysis with ANOVA showed significant differences in AWV/pTLC (*p* = 0.00001), rLV/pTLC (*p* = 0.00001), and AWV% (*P* = 0.000001) among different GOLD spirometric grade

Figure 2 shows that the AWV/pTLC decreased (A), rLV/pTLC increased (B) (both *p* < 0.001 by ANOVA), and the AWV% decreased as the Global Initiative for Chronic Obstructive Lung Disease (GOLD) spirometric grade increased (C)(*p* < 0.001). The AWV% was also lower in symptomatic (CAT score≧10) subjects than non-symptomatic subjects (0.50 ± 0.12% and 0.55 ± 0.15%, respectively, *p* = 0.036, Additional file 1: Figure S2).

*Ai* luminal area of subsegmental airways, *pTLC* predicted TLC, *rLV* right lung volume, *AWV* airway volume, *AWV%* airway volume percent, *LAV%* low attenuation volume percent, *FEV*_*1*_ forced expiratory volume in 1 s, *RV/TLC* residual volume / total lung capacity, *D*_*LCO*_ diffusion capacity. ^a^ and ^b^ indicate *p* < 0.05 and *p* < 0.005, respectively

**Fig. 3:**
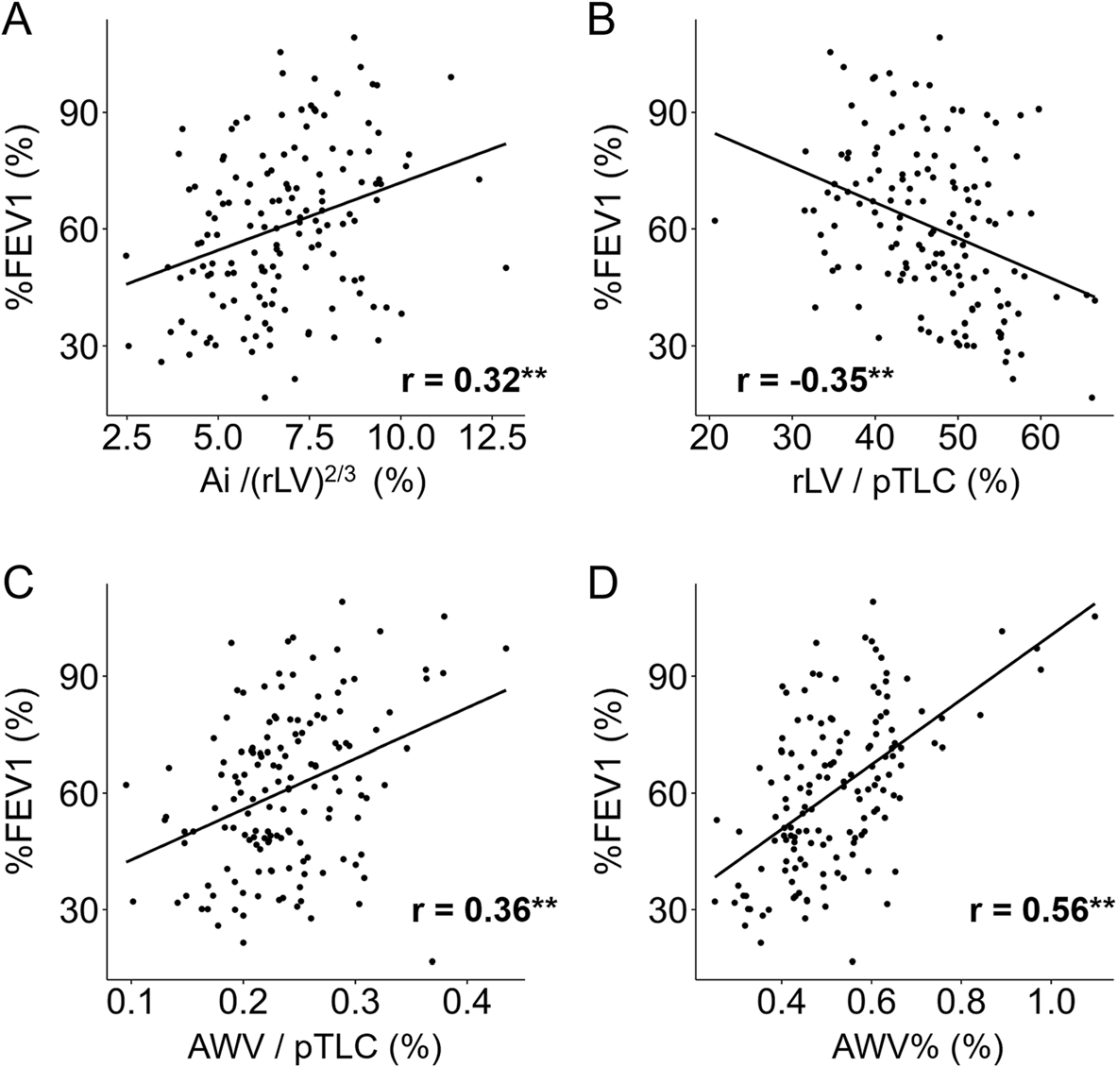
Associations of airway size, lung volume, and airway to lung volume ratio with airflow obstruction. **a** Percentage ratio of lumen area of subsegmental airways (Ai) to cubic root squared right lung volume (rLV), **b** percentage ratio of rLV to predicted total lung capacity (pTLC), **c** percentage ratio of airway volume (AWV) to pTLC, and (**d**) airway volume percent (AWV%) were correlated with percentage of predicted forced expiratory volume in 1 s (%FEV_1_). r value indicates the Pearson correlation coefficient. ** indicates *p* < 0.005

The Pearson correlation tests (Table 2 and Fig. 3) showed that Ai, Ai/(pTLC)^2/3^, Ai/(rLV)^2/3^, rLV, rLV/pTLC, AWV, AWV/pTLC, and AWV% as well as LAV% were all correlated with FEV_1_, %FEV_1_, and RV/TLC. Based on the correlation coefficients, the extent of the correlations in AWV% was higher than in the other measures of the airway and lung sizes, and similar to that in LAV%. Moreover, Table 2 and Additional file 1: Figure S3 show that LAV%, but not AWV%, was associated with %D_LCO_ and D_LCO_/V_A_ while both the indexes were correlated with %TLC. Additional file 1: Figure S4 shows associations of AWV% with TAC, WA%, and LAV%.

**Table 2:** Pearson correlation coefficients between airway and lung size indices, emphysema severity, and physiological measurements

	FEV_1_ (L)	%FEV_1_ (%)	RV/TLC (%)	%D_LCO_ (%)
Lumen area				
Ai (mm^2^)	0.27^b^	0.24^b^	−0.24^b^	− 0.05
Ai / (pTLC)^2/3^ (%)	0.16^a^	0.18^a^	−0.19^a^	−0.09
Ai / (rLV)^2/3^ (%)	0.31^b^	0.32^b^	−0.33^b^	0.01
Lung volume				
rLV (ml)	−0.14	−0.21^a^	0.22^a^	−0.12
rLV / pTLC (%)	−0.37^b^	−0.35^b^	0.33^b^	−0.21^a^
Airway tree volume				
AWV (ml)	0.41^b^	0.43^b^	−0.35^b^	0.00
AWV / pTLC (%)	0.27^b^	0.36^b^	−0.29^b^	−0.06
AWV% (%)	0.48^b^	0.56^b^	−0.47^b^	0.07
Emphysema				
LAV%	−0.48^b^	−0.48^b^	0.43^b^	−0.62^b^

In multivariate analyses (Table 3), AWV% was significantly associated with %FEV_1_ and RV/TLC independent of LAV%, WA%, and TAC. Additional file 1: Table S1 shows that the multivariate model including both AWV% and LAV% as independent variables accounted for more variations of %FEV_1_ and RV/TLC (R^2^ = 0.47 and 0.38, respectively) compared to the models including either AWV% (R^2^ = 0.34 and 0.34, respectively) or LAV% (R^2^ = 0.21 and 0.22, respectively).

**Table 3:** Multivariate linear regression analysis to explore the associations between CT indices and pulmonary function

Model 1 (%FEV_1_)^b^		β^a^	VIF	*p* value
	LAV%	-0.50	2.3	< 0.00001
	WA%	-0.26	1.6	0.73
	TAC	0.21	3.2	0.048
	AWV%	0.35	3.6	0.003
Model 2 (RV/TLC)^b^		β^a^	VIF	p value
	LAV%	0.24	2.3	0.01
	WA%	-0.17	1.6	0.98
	TAC	-0.17	3.2	0.14
	AWV%	-0.28	3.6	0.02

*FEV*_*1*_ forced expiratory volume in 1 s, *RV/TLC* residual volume / total lung capacity, *LAV%* low attenuation volume percent, *WA%* wall area percent, *TAC* total airway count, and *AWV%* airway volume percent. β^a^ indicates standardized β. ^b^ adjusted by age, BMI, pack-years, and CT-measured total lung volume. VIF indicates variance inflation factor

## Discussion

This is the first study to assess the interaction of three-dimensionally measured airway tree and lung volumes on lung function and symptoms in COPD. Although many CT studies have shown that small lumen area of the airways and large lung volume are major features of COPD and physiological studies have emphasized the importance of the relationship between airway and lung size in terms of structure-function, little attention has been paid to airway to lung volume ratio in COPD. Therefore, the present study is important as it established the AWV% as a novel CT biomarker in COPD by showing that a reduction of AWV% is significantly associated with lower FEV_1_ and higher RV/TLC independent of previously reported CT indices such as WA%, LAV%, and TAC. This suggests that a disproportionally small airway tree for a relatively large lung can be a major determinant of airflow obstruction and gas trapping.

The present finding that AWV% is more closely correlated with %FEV_1_ and RV/TLC than simple measurement of lumen area, airway tree volume, and lung volume, as well as the ratio of lumen area to the cubic root squared right lung volume (Table 2), indicates the advantage of this new CT index. These results also confirm the concept that not only each structural change of airway and lung but also the interaction between these factors determines lung function. Because the visibility of airways on CT varies among individuals, it can be challenging to compare the lumen areas of spatially matched airways [36]. Thus, our findings suggest that the use of airway tree volume is more reasonable for assessment of airway size than that of lumen area.

The demographic data showing that %D_LCO_ was 53 ± 14% and LAV% was 29 ± 9% suggest that the majority of the subjects had emphysema in this study. Parenchymal destruction of emphysema could increase lung volume through loss of elastic recoil and induce thoracic gas compression during forced expiration on spirometry [37]. Thus, the increased lung volume due to emphysema might have partially accounted for the close association between decreased AWV% and FEV_1_.

In addition, considering that emphysema can be one of the causes of the airway volume reduction due to loss of alveolar attachments to the outer wall of the small airways [21, 22], one would hypothesize that lower AWV% may be closely associated with higher LAV%. However, the hypothesis is not well supported by the present data that showed the weak association between AWV% and LAV% (*r* = − 0.24) and no association between AWV% and diffusion capacity. This might be because in addition to the impaired airway-parenchymal tethering due to emphysema, the luminal narrowing due to the mucus occlusion, bronchoconstriction, and wall remodeling may also contribute to the reduction in the airway volume measured on CT.

The results of multivariate analysis show that AWV% was associated with airflow limitation and gas trapping independent of LAV%, WA%, and TAC. This finding suggest that in terms of assessment of the structure-function relationship, measuring the airway tree to lung volume ratio is complementary to measuring emphysematous change, impaired visibility of the airways and wall remodelling of the visible central airways, which are established CT findings in COPD lungs [14–17, 38].

The univariate and multivariate analysis (Table 2 and Table 3) show that both AWV% and LAV% had greater impacts on %FEV_1_ and RV/TLC than the other CT indexes. Furthermore, the statistical model including both AWV% and LAV% accounted for more variation of %FEV_1_ (*R*^2^ = 0.47) than the models including either AWV% (*R*^2^ = 0.34) or LAV% (*R*^2^ = 0.21). While emphysema is visually and quantitatively evaluated in clinical setting, the presence and severity of emphysema is not directly linked to airflow obstruction [39, 40], which is measured on spirometry for diagnosis of COPD. Thus, the present findings propose the potential clinical implication of AWV% by showing that combining AWV% to LAV% may allow more accurate estimation of airflow obstruction than emphysema measurement alone. Furthermore, because AWV% reflects both airway dimension and lung volume, and bronchodilation and lung deflation are main targets for standard therapy using long-acting bronchodilators in COPD [41], AWV% might sensitively detect an effect of bronchodilator therapy. A future research is needed to test this possibility.

Abnormal lung growth has recently been proposed as a major contributor to COPD development [26]. Studies have shown that a variability in associations between airway and lung size due to dysanaptic lung growth in early life is associated with lung function [24, 25], and an increase in lung size without change in airway size due to chronic hypoxia at higher altitude is associated with poor lung function [9]. These findings raise the possibility that the present finding that lower AWV% is correlated with lower FEV_1_ and higher RV/TLC might have been affected by abnormal lung growth. Whether the combined presence of smaller airways and larger lung is associated with the early onset of COPD should be examined in a future study.

With respect to clinical symptoms, the present study showed that the AWV% was significantly lower in subjects with a CAT score ≥ 10 than in those with a CAT score < 10. We also performed multivariate logistic regression analysis to test whether a decrease in AWV% was associated with the presence of symptoms after adjusting %FEV_1,_ age, BMI, smoking status, and CT-measured total lung volume but could not detect significant contribution of the AWV% to symptoms (data not shown). Because clinical symptoms are influenced by multiple factors including not only airway disease but also emphysema and systemic comorbidities, such as cardiovascular disease and depression [42], a larger sample size would be necessary to clarify the relationship between the decreased AWV% and symptoms. It might also be useful to apply a specific questionnaire for respiratory symptoms, such as Cough and Sputum Assessment Questionnaire (CASA-Q) [43].

Some limitations need to be described. First, because the lumen size is underestimated in smaller airways due to the resolution of CT [12, 44] and measurement of the AWV% is based on the volumes of all visible airways, including these smaller airways, the AWV% might be underestimated compared to the actual ratio of the airway volume to the lung volume. However, we think that the use of AWV% is valid because the influence of this underestimation should be equally applied in all CT scans. Moreover, the fact that all the CT scans were obtained with one CT scanner under one condition increases the validity of the results. Second, the present study did not use both lungs for measurement of the AWV% because cardiac motion artefacts could increase the measurement error for airway volume in the left lung. Finally, because all the included patients were male, and since the size of airways and extent of airway remodelling differ between males and females [45, 46], whether the present findings are applicable to female patients is unclear.

## Conclusions

This study established a novel CT marker to evaluate airway tree to lung volume ratio, AWV%, and showed that a disproportionately small airway tree volume for a given lung volume is associated with worsening of airflow obstruction and gas trapping. This index is a promising CT biomarker that is easily measured and allows for improved understanding of the interaction between airway disease and lung hyperinflation on lung function and clinical outcomes in COPD.

## Additional file

Additional file 1: **Figure S1** Examples of original CT images and segmentations of airway trees and all branches in the right lung. **Figure S2** The airway volume percent in symptomatic and non-symptomatic subjects. **Figure S3** Associations of the airway volume percent and low attenuation volume percent with physiological measurements. **Figure S4** Associations of the airway volume percent with currently established CT indexes. **Table S1** Multivariate regression models regarding pulmonary function. (PDF 514 kb)

## Abbreviations

Ai: Airway lumen area
AWV: Airway volume
AWV%: Airway volume percent
CAT: COPD Assessment Tests
COPD: Chronic obstructive pulmonary disease
CT: Computed tomography
CT-TLV: Total lung volume measured on CT
D_LCO_: Diffusion capacity
FEV_1_: Forced expiratory volume in 1 s
HU: Hounsfield units
LAV%: Low attenuation volume percent
pTLC: Predicted total lung capacity
rLV: Right lung volume
RV/TLC: Residual volume/total lung capacity
TAC: Total airway count
