# Supplementary material for: Associations of airway tree to lung volume ratio on computed tomography with lung function and symptoms in chronic obstructive pulmonary disease
Source: Respir Res. 2019 Apr 18;20:77. doi: 10.1186/s12931-019-1047-5 (PMC6471860; doi:10.1186/s12931-019-1047-5)
Supplement: Supplementary file 1 — Figure S1 Examples of original CT images and segmentations of airway trees and all branches in the right lung. Figure S2 The airway volume percent in symptomatic and non-symptomatic subjects. Figure S3 Associations of the airway volume percent and low attenuation volume percent with physiological measurements. Figure S4 Associations of the airway volume percent with currently established CT indexes. Table S1 Multivariate regression models regarding pulmonary function. (PDF 514 kb) [file 12931_2019_1047_MOESM1_ESM.pdf]

Online supplement

Associations of airway tree to lung volume ratio on computed tomography with lung function and symptoms in chronic obstructive pulmonary disease

Naoya Tanabe [1], Susumu Sato [1], Tsuyoshi Oguma [1], Hiroshi Shima [1], Atsuyasu Sato [1], Shigeo Muro [1], Toyohiro Hirai [1]

[1] Department of Respiratory Medicine, Graduate School of Medicine, Kyoto University, 54 Kawahara-cho, Shogoin, Sakyo-ku, Kyoto 606-8507, Japan

List

- **Figure S1. Examples of original CT images and segmentations of airway trees and all branches in the right lung**
- **Figure S2. The airway volume percent in symptomatic and non-symptomatic subjects.**
- **Figure S3. Associations of the airway volume percent and low attenuation volume percent with physiological measurements**
- **Figure S4. Associations of the airway volume percent with currently established CT indexes**
- **Table S1. Multivariate regression models regarding pulmonary function**

Figure S1. Examples of original CT images and segmentations of airway trees and all branches in the right lung

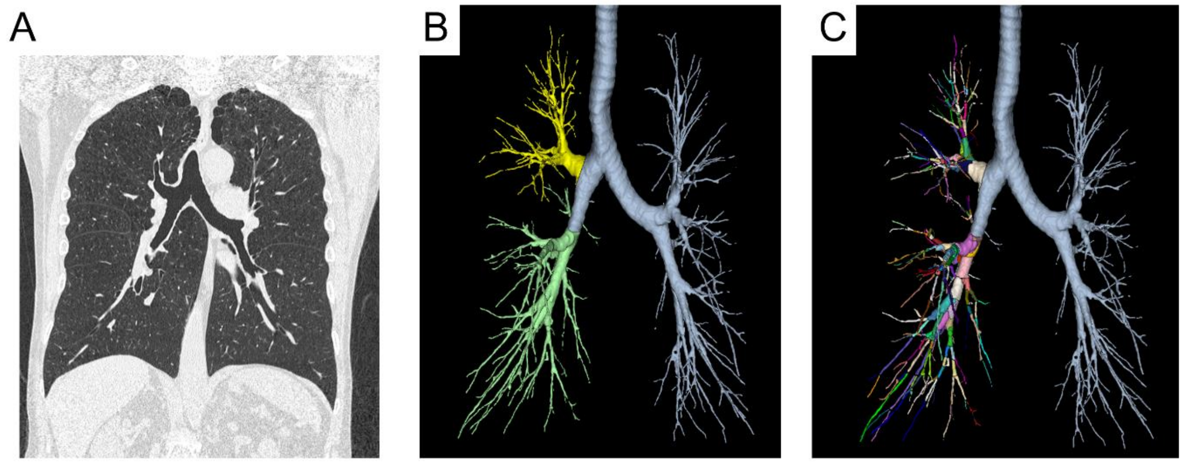

The airway tree was automatically segmented from the original CT image (A), and the portions of the right upper and middle-lower lobes (RUL and RMLL) were extracted (B, red and green parts). (C) The total airway count (TAC) was also measured by labelling all branches in the tree.

Figure S2. The airway volume percent in symptomatic and non-symptomatic subjects.

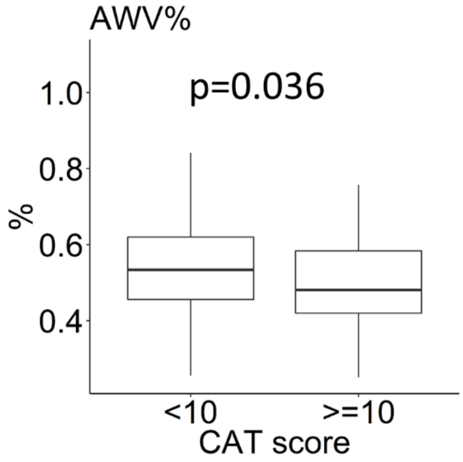

Symptoms were assessed with the COPD assessment test (CAT score). The airway volume percent (AWV%) in symptomatic (CAT≧10) was lower than non-symptomatic (CAT<10) subjects.

Figure S3. Associations of the airway volume percent and low attenuation volume percent with physiological measurements

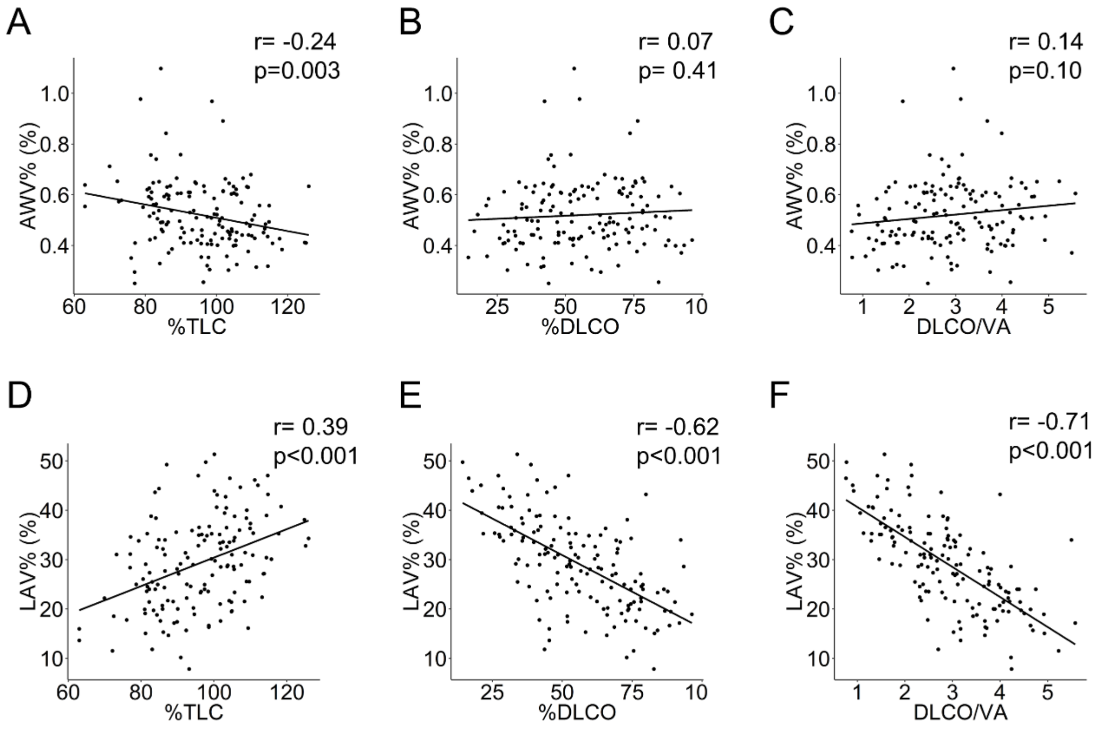

The airway volume percent (AWV%) was correlated with (A) percent total lung capacity (%TLC), but not with (B) percent diffusing capacity of the lung for carbon monoxide (%D_{LCO}) and ratio of diffusing capacity to alveolar ventilation (D_{LCO}/V_A). The low attenuation volume percent (LAV%) was correlated with (D) %TLC, (E) %D_{LCO}, and (F) D_{LCO}/V_A.

Figure S4. Associations of the airway volume percent with currently established CT indexes

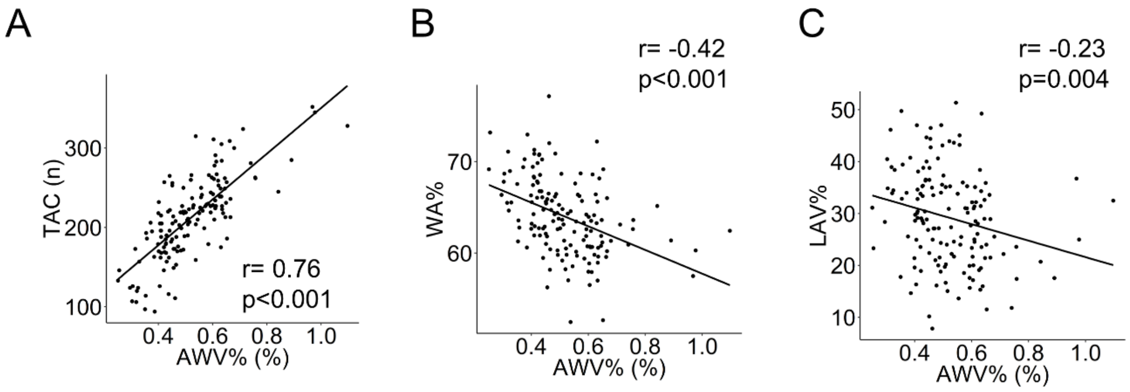

The airway volume percent (AWV%) was correlated with the (A) total airway count (TAC), (B) wall area percent (WA%) for sub-segmental airways, and (C) low attenuation volume percent (LAV%).

Table S1. Multivariate regression models regarding pulmonary function

%FEV_1		CT measures	R^2	AIC
	Model 1	LAV%	0.21	389
	Model 2	AWV%	0.34	364
	Model 3	LAV%+AWV%	0.47	332
**RV/TLC**		**CT measures**	**R^2**	**AIC**
	Model 1	LAV%	0.22	388
	Model 2	AWV%	0.34	362
	Model 3	LAV%+AWV%	0.38	355

FEV_1 = forced expiratory volume in 1 second, RV/TLC = residual volume / total lung capacity, LAV% = low attenuation volume percent, and AWV% = airway volume percent. Multivariate linear regression models to estimate %FEV_1 and RV/TLC were made by using (1) LAV%, (2) AWV%, and (3) both LAV% and AWV%. All models were adjusted by age, BMI, pack-years, and CT-measured total lung volume. R^2 = adjusted R-squared. AIC = Akaike information criterion to compare quality of different models, where lower AIC indicates better model.
